# Revisiting the 2015 MDS diagnostic criteria for Parkinson disease: insights from autopsy-confirmed cases

**DOI:** 10.1038/s41531-025-01206-6

**Published:** 2025-12-13

**Authors:** Susan H. Fox, Daniel G. Luca, Ronald B. Postuma, Roongroj Bhidayasiri, Francisco Cardoso, Gabor G. Kovacs, Regina Katzenschlager, Claudia Trenkwalder

**Affiliations:** 1 University of Toronto, Movement Disorder Clinic, Edmond J Safra Program in Parkinson Disease, Toronto Western Hospital, Toronto, ON Canada; 2 Department of Neurology, Washington University in St. Louis, St. Louis, MO USA; 3 The Neuro (Montreal Neurological Institute-Hospital), McGill University, Montreal, QC Canada; 4 Centre for Advanced Research in Sleep Medicine, Hôpital du Sacré-Coeur de Montréal, Montréal, QC Canada; 5 Chulalongkorn Centre of Excellence for Parkinson’s Disease & Related Disorders, Department of Medicine, Faculty of Medicine, Chulalongkorn University and King Chulalongkorn Memorial Hospital, Thai Red Cross Society, Bangkok, Thailand; 6 The Academy of Science, The Royal Society of Thailand, Bangkok, Thailand; 7 Movement Disorders Unit, Neurology Service, Internal Medicine Department, The Federal University of Minas Gerais, Belo Horizonte, Brazil; 8 Tanz Centre for Research in Neurodegenerative Diseases, University of Toronto, Toronto, ON Canada; 9 Edmond J. Safra Program in Parkinson’s Disease and the Morton and Gloria Shulman Movement Disorders Clinic, Toronto Western Hospital, Toronto, ON Canada; 10 Krembil Brain Institute, University Health Network, Toronto, ON Canada; 11 Department of Laboratory Medicine and Pathobiology, University of Toronto, Toronto, ON Canada; 12 Laboratory Medicine Program, University Health Network, Toronto, ON Canada; 13 Department of Neurology and Karl Landsteiner Institute for Neuroimmunological and Neurodegenerative Conditions, Klinik Donaustadt, Vienna, Austria; 14 Paracelsus-Elena Hospital, Center for Parkinson and Movement Disorders, Kassel, Germany; 15 Dept. Neurosurgery, University Medical Center Goettingen, Goettingen, Germany

**Keywords:** Diseases, Medical research, Neurology, Neuroscience

## Abstract

The 2015 International Parkinson and *Movement Disorder* Society (MDS) Diagnostic criteria for Parkinson Disease are based on expert consensus opinion and defines core motor features, ‘Absolute Exclusion Criteria’ and a balance of ‘Supportive Criteria’ and ‘Red Flags’. To assess validity of each criterion in pathologically-confirmed cases, a scoping literature review between 1988-2024 using search terms for clinicopathological PD and atypical parkinsonian disorders identified 28 articles. Supportive criteria were higher in PD, with excellent levodopa response and rest tremor most useful. Absolute exclusion criteria and red flags were present more often in atypical parkinsonian disorders. However, supranuclear gaze palsy, rapid progression of gait impairment to wheelchair requirement and bilateral symptoms were reported in >5% PD. Data was limited by few appropriate pathological studies with sufficient clinical data; challenges in applying highly-specific definitions to retrospective studies and likely co-pathologies. This review provides empiric data to support some items of the MDS Criteria with future potential refinement.

## Introduction

Making a diagnosis of Parkinson Disease (PD) currently relies on clinical history and skilled neurological examination, performed by medical professionals with sufficient training in neurology. Differentiation of PD from atypical Parkinsonian disorders such as Dementia with Lewy Bodies (DLB), Multiple System Atrophy (MSA), Progressive Supranuclear Palsy (PSP and Corticobasal Degeneration (CBD) may be difficult, especially in the early disease stages. Recent efforts have proposed integrating new biological markers of alpha-synuclein with genetics and imaging to improve earlier differentiation of biological disease entities^1,2^. These biomarkers, including seeding amplification assays (SAA) for alpha -synuclein in CSF, skin or possibly blood, need careful validation regarding sensitivity and specificity. Importantly, at the time of writing this article, access to SAA and other tests is not widespread and mostly limited to research studies^3^. For practicing clinicians, a clinical diagnosis will remain the first step, aided by available imaging or laboratory tests depending on region and health care system^3^.

The first widely accepted clinical definition of PD, the Queen Square Brain Bank criteria (UK Parkinson’s Disease Society Brain Bank criteria), originated from a clinico-pathological study. This included bradykinesia as the key symptom, with at least one of rest tremor, rigidity and/or unexplained postural instability^4,5^. These criteria did not include genetic anchors and patients with a marked positive family history of PD were excluded from PD diagnosis. An appraisal of these clinical criteria was published in 2003 by the Scientific Issues Committee (SIC) of the International Parkinson and Movement Disorders Society (MDS)^6^. The authors highlighted some shortcomings and the need to use ancillary testing. After several years, the MDS started an initiative to develop revised criteria for clinical PD, which were published in 2015^7^. Unlike the Queen Square Brain Bank criteria, the MDS clinical diagnostic criteria were not based on clinico-pathological correlations but on a literature review and a consensus based expert opinion. The anchor of the MDS criteria was expert clinical diagnosis; that is, the criteria were designed to mimic and codify the diagnostic process of internationally recognized clinical experts.

In the 2015 MDS criteria, the primary definition for use in clinical practice is termed “Clinically Probable PD”. This requires a parkinsonian syndrome (bradykinesia plus at least one feature of rest tremor and rigidity), absence of “absolute exclusion criteria”, and a balance between positive and negative features of the disease (that is, allowing “Red Flags” as long as they are balanced by “supportive criteria”). The more restrictive definition of “Clinically-Established PD” requires an absence of absolute exclusion criteria, at least two supportive criteria, and no Red Flags. Absolute Exclusion Criteria were defined as negative features that are highly specific for an alternative diagnosis, but which may occur in <3% of ‘true’ PD^7^. Red Flags were described as negative features which are potential signs of alternate pathology, but with lower or uncertain specificity. Red Flags rule out probable PD only when they cannot be counterbalanced by supportive criteria; that is, the number of red flags must not exceed the number of supportive criteria, and no more than 2 red flags are allowed.

A clinical validation of these criteria showed 93% overall accuracy (89% sensitivity and 95% specificity) of the Clinically-Probable PD criteria, compared to 86% accuracy of Queen Square Brain Bank criteria (89% sensitivity, 79% specificity), tested against the gold standard of expert clinical diagnosis (neurologists with >10 y experience in PD diagnosis)^8^. A recent autopsy validation of the clinically-probable PD criteria using the UK Brain Bank material in 141 PD and 126 non-PD parkinsonism found an overall accuracy of 92.5% for the final criteria evaluation at death and 89.5% for the criteria at initial clinical evaluation^9^. However, this study did not evaluate the clinic-pathological accuracy of the individual items (ie each of the Supporting Criteria, Absolute exclusion criteria and Red Flags) in the MDS Clinical diagnostic criteria, but rather evaluated the clinical application of the MDS criteria as a whole. It also focused on the first 5 years and the final stages of PD. Moreover, although the 2015 MDS Criteria show good accuracy in research studies, where experts have taken time to carefully review the criteria, their complexity may pose challenges for non-expert clinicians, especially in clinical environments with limited access to specialized training or resources. Refining the current MDS criteria to the essential and most accurate features may improve utility for the practising clinician.

We conducted a scoping literature review from 1988, (the year of publication of the Queen Square Brain Bank Criteria), to 2024 to study the utility of each individual item of the 2015 MDS Clinical diagnostic criteria for PD in studies when the final pathological diagnosis had been confirmed. Such an appraisal may help refine, update, and optimise the MDS clinical criteria. The aim was to improve sensitivity and specificity of the diagnosis of PD while highlighting the obstacles of applying clinical diagnostic criteria in a real-world setting.

## Results

Sixty articles were initially identified. Thirty-two were excluded due to lack of sufficient information regarding clinical data, leaving twenty-eight papers included in the final review.

The mean percentage of subjects (and range) reported for each supportive criterion, absolute exclusion criterion and red flags as determined in each pathologically confirmed disorder were calculated. For PD the accumulated total was *n* = 1512 subjects. Accumulated total of *n* = 1177 subjects with atypical parkinsonian syndrome included *n* = 45 DLB, *n* = 806 MSA, *n* = 276 PSP and *n* = 50 CBD (Table 1). Sensitivity and specificity estimates are presented in Supplementary Material Table 2.

**Table 1:** Percentage of pathologically confirmed PD, DLB, MSA, PSP and CBD with each item of MDS Diagnostic Criteria

	Clinicopathological diagnosis mean% (range)				
Supportive Criteria	PD	DLB	MSA	PSP	CBD
Clear and dramatic beneficial response to dopaminergic therapy. During initial treatment patient returned to normal or near normal.	84.1 (69–100)^10,11,14,27,28–32^ *n* = 917	ND	35.9 (16–54)^10,31–35^ *n* = 293	15 (7–23)^10,14^ *n* = 63	NA
Marked improvement with dose increases or marked worsening with dose decreases (Objective eg > 30% UPDRS III with change of treatment) or subjective from history). Or unequivocal and marked on/off with predictable end-of-dose wearing off. Fluctuations	57.1 (33–84)^10,27,31,35–38^ *n* = 788	ND	29 (24–39)^10,31,35^ *n* = 266	36^10^ *n* = 18	NA
Presence of levodopa-induced dyskinesia(s)	61.7 (55– 80.8)^10,27,31,38,39^ *n* = 560	NA	36.4 (27–39.5)^10,31,34,35^ *n* = 289	28^10^ *n* = 18	NA
Rest tremor of a limb (in past or current examination)	68 (43–91)^11,14,27,30–32,37,38,40,41^ *n* = 675	39^41^ *n* = 39	28 (26–30)^32,41^ *n* = 256	22 (7–37)^14,41^ *n* = 45	NA
Presence of olfactory loss	94.6^11^ *n* = 39	NA	NA	NA	NA
Presence of Cardiosympathetic denervation (MIBG SPECT)	ND	ND	ND	ND	ND

Absolute Exclusion Criteria	PD	DLB	MSA	PSP	CBD
Cerebellar /abnormalities/ Symptoms					
Gait Ataxia	NA	11^41^	36 (23–49)^35,41^	43^41^	ND
Limb Ataxia	NA	6^41^	47.5 (47–48)^35,41^	43^41^	ND
Non-Specific Ataxia	1 (0–1)^31,32^ *n* = 134	ND *n* = 18	47.9 (32–64)^31,32,42^ *n* = 231	ND *n* = 14	ND
Downward Supranuclear gaze palsy or selective slowing of downward vertical saccades (*no differentiation of direction)	6.8*^15^ *n* = 146	ND	20.6^43^ Downgaze only 21.5^43^ *n* = 160	66.9 (61–75)^15,16,43^ Downgaze only 37.5^43^ *n* = 51	ND
Behavioural Variant frontotemporal dementia /progressive aphasia	ND	NA	19 (13–25)^42,43^ *n* = 176	8 (8)^16,43^ *n* = 37	ND
Parkinsonian features restricted to lower limbs for more than 3 years	ND	ND	ND	ND	ND
Absence of observable response to high dose levodopa despite at least moderate severity of disease	1.7 (0–2.7)^14,28,44^ *n* = 247	ND	29 (27–31.2)^33,44^ *n* = *27*	75^44^ *n* = *16*	ND
Unequivocal Cortical sensory loss; limb apraxia; progressive aphasia	ND	ND	ND	ND	33^16^
Normal functional of the presynaptic dopaminergic system	0^12^ *n* = 47	NA^12^	2.4^12^ *n* = 42	6.8^12^ *n* = 73	40^12^ *n* = 10

Red Flags	PD	DLB	MSA	PSP	CBD
Rapid progression of Gait impairment requiring wheelchair within 5 years of disease onset	8.7 (3–14.4)^28,38^ *n* = 266	7^45^ *n* = 14	30.1 (13–53)^43,45^ *n* = 165	33 (20–46)^43,45^ *n* = 37	8^45^ *n* = 13
Complete lack of progression within 5 years, unless related to treatment	0.8%^14^ *n* = 116	NA	ND	ND	ND
Early Bulbar dysfunction, severe dysphonia or dysarthria (speech unintelligible most of the time or severe dysphagia (requiring soft food, NG or PEG within first 5years)	2.5^14^ *n* = 116	ND	46^43^ *n* = 160	23^43^ *n* = 13	ND
Inspiratory stridor	0.8^14^ *n* = 116	NA	21 (10–31.8)^35,43^ *n* = 363	0^35^ *n* = 13	ND
Severe autonomic failure within first 5 years with Orthostatic hypotension (see definition)	4.7 (0–9.4)^14,32^ *n* = *151*	NA	47 (18 – 68.7)^14,32,33,35,44^ *n* = 438	0^44^ *n* = 16	ND
Severe urinary retention /incontinence (associated with erectile dysfunction in men)	0^46^ *n* = 11	15^46^ *n* = 14	46.3 (31–87)^31,33,43,46,47^ *n* = 246	45^46^ *n* = 13	ND
Recurrent falls (>1 per year) within first 3 years	3.8 (0–7.7)^14,41^ *n* = 124	28^41^ *n* = 19	28.8 (13–42.5)^41,43,47^ *n* = 274	33.6 (29–38.5)^41,43^ *n* = 28	22^16^ *n* = 27
Disproportionate (dystonic) anterocollis or contractures of hands/feet within first 10 years	NA	NA	21 (10–32.5)^32,35^ *n* = 380	15.4^32^ *n* = 17	ND
Absences of common non-motor symptoms: despite 5 years of disease duration: sleep disorder: autonomic dysfunction, hyposmia, psychiatric symptoms	ND	ND	ND	ND	ND
Otherwise, unexplained Pyramidal tract signs	7 (6.9–7)^14,31^ *n* = 108	11^41^ *n* = 18	40.9 (31.3–52)^31,32,35,37,41,42^ *n* = 536	18.6 (14–23.1))^32,41^ *n* = 31	ND
Bilateral symmetric Parkinsonism	17.6 (0.02–28)^14,31,44^	NA	33.9 (24–43.7)^31,44^	84 (81–87)^16,44^	48^16^

*ND* not done/no data, *NA* not sufficient data for analysis, *PD* Parkinson Disease, *DLB* Dementia with Lewy Bodies, *MSA* multiple system atrophy, *PSP* progressive supranuclear palsy, *CBD* Corticobasal degeneration, *NG* Nasogastric tube, *PEG* Percutaneous endoscopic tube.

### Supportive criteria

A beneficial response to levodopa was determined in 84.1% of 917 accumulated PD cases; compared to 35.9% in 293 MSA and 15% in 63 cases of PSP. There was a lack of consistent information on the timing of these levodopa responses in relation to disease duration. Variability in the clinical descriptions of levodopa response was noted; most studies did not report the amplitude of levodopa response, which is a critical component of the MDS criteria. Only one study documented objective rating scale changes^10^, which reported marked improvement (>10 point change in UPDRS III) among 68% of PD; 16% of MSA and 16.7% of PSP. Fluctuations in levodopa response, including wearing-off, were noted in 59% of 788 PD patients compared to 29% of 266 MSA and 36% of 18 PSP cases. Levodopa induced dyskinesia (without further descriptions of the dyskinesias) was reported in 61.8% of 560 PD, 36.4% of 289 MSA and 28% of 18 PSP cases. No data was available for DLB or CBD. Rest tremor was noted in 71% of 675 PD cases and 39% of 39 DLB cases, compared to 28% of 256 cases of MSA and 22% of 45 PSP cases. Many studies reported presence of ‘tremor’ without specifying ‘rest’ tremor; these estimates were omitted from analysis. Only one autopsy study investigated olfaction using objective UPSIT scores, which noted olfactory loss in 94% of 39 PD cases^11^. There was no data on olfactory loss for DLB, or any atypical parkinsonian disorder. No studies were found reporting MIBG imaging.

### Absolute exclusion criteria

Cerebellar abnormalities were described in variable terms, including gait ataxia, limb ataxia or non-specific ataxia. In 134 PD subjects, 1% had documented ataxia. In 18 cases of DLB, 11% had limb ataxia and 6% had gait ataxia. In 231 cases of MSA, 47.9% had non-specific ataxia, 36% had gait ataxia and 47.5% limb ataxia. In 14 PSP cases, limb and gait ataxia were both noted in 43% of cases. No data was available for ataxia in CBD. Supranuclear gaze (SNG) palsy was rarely specified as ‘downward vertical gaze palsy or selective slowing of downward vertical gaze’ as per 2015 MDS Criteria. In 146 PD subjects, 6.8% were noted to have a SNG palsy but no direction was specified. Out of 160 MSA cases, 20.6% had a SNG palsy, with downgaze specified in 21.5% of MSA subjects with SNG palsy (7 cases, 4.37% of all MSA cases). In 51 PSP subjects, 66.9% exhibited SNG palsy, with downgaze specifically noted for only 13 subjects (37.5% of PSP cases). No data was available for DLB or CBD.

‘Behavioural variant frontotemporal dementia or primary progressive aphasia within 5 y of diagnosis’ was rarely reported, and no formal cognitive assessments were included. In 176 MSA cases, 19% had frontal lobe disturbance (i.e., without necessarily having clear dementia), along with 8% of 37 PSP cases. No data was available for PD, DLB or CBD. ‘Absence of an observable response to levodopa despite moderate disease severity’ was recorded in 1.7% of 247 PD cases, 29% of 27 MSA cases and 75% of 16 PSP cases. No data was available for DLB or CBD. ‘Unequivocal cortical sensory loss’ was only reported in CBD in 33% of 27 cases; no data was available for any other disorder. ‘Normal functional neuroimaging of the presynaptic dopaminergic system’ was reported in one study, with normal scans reported in 0% of 47 PD cases, 2.4% of 42 MSA cases, 6.8% of 73 PSP cases and 40% of 10 CBD cases. ‘Parkinsonian features restricted to the lower limbs for more than 3 y’ were not assessed in any study. Drug-induced parkinsonism and ‘documentation of an alternative condition known to produce parkinsonism’ were not included in data collection.

### Red Flags

‘Rapid gait impairment to wheelchair requirement within 5 years’ was reported in 8.7% of 266 PD and in 7% of 14 DLB subjects. By contrast, the rate was 30.1% of 165 MSA, 33% of 37 PSP and 8% of 13 CBD cases. ‘A lack of progression over 5 y unrelated to treatment’ was noted in 1 out of 116 PD cases (0.8%); no data was available for any other disease category. Bulbar dysfunction was recorded as severe in the clinical description in 2.5% of 116 PD subjects; with no data for DLB. For MSA, 46% of 160 and in PSP, 23% of 13 cases were reported, with no data for CBD. Inspiratory dysfunction was noted in 0.8% of 116 PD and 0% in 13 PSP compared to 21% of 363 MSA. ‘Severe autonomic failure in the first 5 y resulting in orthostatic hypotension’ was reported in 4.7% of 151 PD cases; compared to 47% of 438 MSA and 0% of 16 PSP. There was no data for DLB or CBD. ‘Severe urinary dysfunction in the first 5 y’ was reported in 0% of 11 PD; 15% of 14 DLB; compared to 46.3% of 246 MSA and 45% of 13 PSP cases. No data was reported for CBD. ‘Recurrent falling because of impaired balance within 3 y’ was reported in 3.8% of 124 PD cases and 28% in 19 DLB cases. In 274 MSA cases, these early falls were reported in 28.8%, in 33.6% of 28 PSP cases and in 22% of 27 CBD cases. ‘Disproportionate/early anterocollis or contractures’ were reported in 21% of 380 MSA and 15.5% of 17 PSP Cases. No data was available for PD, DLB or CBD. ‘Absence of any of the common non-motor features despite 5 y disease duration’ was not reported for any disease. ‘Otherwise unexplained pyramidal signs’ were noted in 7% of 108 PD and 11% of 18 DLB. In 536 MSA cases this was noted in 40.9% and in 31 PSP cases in 18.6%. No data was available for CBD.

## Discussion

This retrospective descriptive scoping review aimed to determine the accuracy of the individual items of the 2015 MDS PD diagnostic criteria using published clinico-pathological studies. This study goes beyond the recent clinicopathological analysis reported by Virameteekul et al.^9^, which validated the overall clinical diagnostic accuracy of the 2015 MDS Criteria as a single, unified tool, in a large, single-centre autopsy cohort. That study also limited the analysis to the first 5 years and the final disease stages. In contrast, our review evaluated a larger and more diverse pathological series across multiple studies and all disease stages, and focused specifically on the performance of each individual diagnostic item. By deconstructing supportive criteria, absolute exclusions, and red flags, our findings highlight both the consistency of the criteria in distinguishing PD from atypical parkinsonian disorders and the limitations of certain items due to vague definitions, inconsistent documentation, and potential overlap with co-pathologies. These results reinforce the validity of the MDS criteria while underscoring the need for refined definitions, temporal clarity, and standardized reporting in future iterations to enhance clinical utility across diverse practice settings.

Overall, the main items of the 2015 MDS diagnostic Criteria for PD were at least numerically consistent, such that supportive criteria were higher in pathologically confirmed PD than MSA and PSP. Absolute exclusion criteria and red flags—when available - were higher in MSA, PSP, DLB and CBD than PD. However, some individual items were less than optimal for diagnosing PD, due to lack of specificity and non-validated definition of some terms that resulted in many non-PD features being reported in PD cases and vice versa.

The supportive criterion of a ‘positive’ levodopa response was more often reported in PD compared to MSA or PSP, consistent with prior knowledge. However, the difference may appear to be less marked than the assumptions upon which the criteria were designed, likely because the descriptions of levodopa response as a “clear and dramatic beneficial response” were usually not recorded. Rather, the descriptions reported were subjective and based on pragmatic documentation in clinical practice. The 2015 MDS criteria posited that levodopa response is only diagnostically useful at the extremes, such that absent response argues against PD and clear/dramatic benefit argues for PD. Equivocal, mild, or even moderate responses to levodopa were not included in the criteria. This needs to be discussed in future refinements of the criteria, as many clinical series report mild or moderate responses. Future refinements to PD clinical diagnostic criteria will need to clarify the description of levodopa-responsiveness, even quantifying a short-term response or including long-term response, and could possibly incorporate technology-based assessments.

Similar to levodopa response, difficulties in documentation of fluctuations limited evaluation. The required ‘30% difference of the UPDRS scores’ was not reflected in most clinicopathological reports. The ‘presence of levodopa-induced dyskinesias’ is a terminology that could easily be retrieved from clinical reports (if documented at all) – and therefore its estimates may be more reliable than those for the term “fluctuations”. However, some subjects with MSA and PSP may also have a levodopa-response with dyskinesia. Therefore, refining this item to emphasize duration and phenomenology of dyskinesia experienced (e.g., excluding orofacial dystonia, which is more common in MSA) may improve the criterion.

For the supportive criterion of ‘rest tremor on examination’, the findings show that rest tremor was reported in PD, but also in DLB, PSP and MSA. ‘Tremors’ were mentioned in many reports, but there was rarely an explicit differentiation between rest tremor and any other forms of tremor. As some patients with PD lack rest tremor, it is diagnostically useful if present but not if absent.

Only two ancillary tests were included in the 2015 MDS Criteria as supportive criteria. Evidence of cardiac sympathetic denervation using MIBG-SPECT was not reported in any series and may reflect limited clinical availability and thus lack of usefulness as an item in the diagnostic criteria. Objective (smell test) loss or reduced olfaction was reported in a high proportion of PD, but no data was available for any atypical parkinsonian disorder group to determine use in differentiating PD.

For Absolute exclusion criteria, the data, where available, showed that a normal DAT scan, the presence of cerebellar ataxia and lack of levodopa response are useful absolute exclusion criteria (occurring in <3% of PD). Functional neuroimaging of the dopaminergic system using dopamine transporter SPECT (DAT) differentiates nigrostriatal dopamine loss from non-degenerative conditions such as essential tremor, drug-induced parkinsonism, dystonia or functional movement disorders. In this review, however, only one study was identified with pathological correlations to imaging studies^12^. No PD cases had normal DAT scan imaging, whereas a few MSA, PSP and particularly CBD reported normal dopamine imaging. This is in keeping with the literature that in rare cases, particularly in CBD, DAT scanning may not distinguish atypical parkinsonian disorders from non-neurodegenerative conditions, whereas its sensitivity for PD (compared to normal controls) is almost uniformly 100%^13^.

The presence of cerebellar ataxia in any form may be a useful exclusion criterion for PD. ‘Unequivocal cerebellar abnormalities” were more commonly described in MSA and PSP although the differentiation between gait, limb and non-specific cerebellar ataxia was not reliably defined. No study reported on cerebellar eye findings.

The “absence of observable response to high dose levodopa” was mentioned in few clinical series but was noted in 75% of pathologically proven PSP patients, and may be a good exclusion criterion for PD. In contrast, as noted above in the supportive criteria, the marked levodopa response (15%) and unequivocal motor fluctuations (36%) in some cases of pathologically confirmed PSP is higher than expected. However, the specific amplitude and characteristics of fluctuations were rarely noted in the autopsy studies – it is likely that some cases with fluctuations would have had modest/equivocal fluctuations or even other variability of clinical features, which would not have met the specific definition laid out in the MDS criteria. Overall, the findings would suggest in clinical practice that about a quarter of PSP cases have levodopa-responsiveness. The levodopa-response may be due to Lewy body co-pathology (PD) in PSP as was noted in a small number of cases 4 /18 cases^10^ and 8 /45 cases^14^. However, correlations between copathology and fluctuations were not assessed in the autopsy studies. None of the series reported the pathological descriptors of subtypes of PSP ie was the parkinsonian subtype more likely to be levodopa-responsive.

The presence of SNG palsy appeared to be less useful as an absolute exclusion criterion. There were a small number of PD patients with SNG palsy, but in the publication^15^ the direction of gaze (“downward”) was not mentioned and thus SNG could be a nonspecific and age-dependent symptom. Using SNG palsy to differentiate PSP from other atypical parkinsonian syndromes was not clear, as cases of MSA were also noted to have a SNG palsy.

The criterion of “presence of probable behavioural variant frontotemporal dementia (FTD)’ was difficult to evaluate, due to lack of complete neuropsychological evaluation and variability in terminology used e.g., ‘frontal lobe disturbance’ ranged from dysexecutive screening scores to behavioural syndromes. No studies in PD reported this feature. Of note, the proportion of MSA cases (19%) was higher than expected, compared to PSP (8%). However, this may simply reflect the variable methods described to define frontal lobe dysfunction between the different centres. Early frontal lobe dysfunction and cognitive impairment is increasingly recognized in MSA and maybe an important clinical evaluation to assist in the differential diagnosis of PD.

As noted in the 2015 MDS Criteria paper, the presence of ‘dementia’ is not an exclusion for PD diagnosis. With increased awareness of cognitive changes in all these parkinsonian disorders, individual cognitive-based criteria may need to be further refined (or excluded as a differential diagnostic criterion).

The exclusion criterion of “Parkinsonian features restricted to lower limbs for more than 3 years” was not documented in any clinical series, probably indicating this is not a feature documented by neurologists in clinics. Likewise, “unequivocal cortical sensory loss” could not be reliably assessed as this sign was not mentioned as explicitly described in the clinical MDS Criteria except in one series of CBD patients^16^.

Red Flags, where reported, were present at a higher proportion in atypical parkinsonian disorders than PD, indicating their usefulness in differentiating these disorders from PD. However, the specific definitions for most Red Flags were difficult to apply at the time of diagnosis, as they are proposed retrospectively with specific time frames (e.g., “Severe autonomic failure in first 5 y of disease”) and there was lack of clear documentation when these features started. With these caveats in mind, the items that appeared to best differentiate PD from atypical parkinsonian disorders (i.e., seen in <2.5% of PD cases) were ‘inspiratory stridor’ in MSA, severe urinary retention /incontinence in MSA, PSP and DLB and early bulbar dysfunction in MSA and PSP.

Items with less clear discriminative value were ‘rapid progression of gait impairment’, ‘recurrent falls’, and ‘bilateral symmetric Parkinsonism’, as these features were reported in 3.8–17.6% of PD cases. This again likely reflects the strict definitions for the timing of the symptom was reported plus lack of clear clinical details. Indeed non-optimized dosing of levodopa may have been a factor in some of these symptoms although no correlation with levodopa dosing and response was reported. Another potential explanation for some cases could have been co-morbidity, particularly in those with advanced age. Items with little or no data were ‘complete lack of progression within 5 years’ and ‘absence of any non-motor features’, reflecting a lack of specific documentation in retrospective series.

An overall limitation related to missing information from retrospective charts is bias from selective documentation, according to specific diagnosis. Thus it is likely that what clinicians write in the chart reflects their clinical thought processes. Missing information can bias estimates, particularly if there are tendencies in one direction. For example, patients with clinically suspected PSP may have careful documentation of features such as extra-ocular movements. By contrast, for those with otherwise classic PD, these features are less likely to be systematically examined and documented, leading to an underestimation of their true occurrence in PD.

Another limitation of this review is that different centres used slightly different pathological definitions, although key diagnostic features were included in all to enable collating data and reviewing clinical information (as reviewed in supplementary Table 1). Of note, most series did not differentiate the clinical features of DLB from PD, as consistent with current disease definitions. Lewy-related pathology is a common neuropathological hallmark of several clinically defined phenotypes including PD, PD with mild cognitive impairment, PDD, and DLB. These cannot be reliably distinguished on neuropathological examination alone. Moreover, the presence of concomitant AD neuropathological change is often seen in cases with cognitive decline (i.e., PDD and DLB)^17^. According to recent neuropathology consensus criteria^18^ the terms PD-MCI, PDD or DLB should not be used to describe neuropathological findings alone. Future clinical diagnostic criteria for PD may need to be more specific around clinical features of cognition. In addition, most studies lacked screening for TDP-43 pathology in the limbic system to detect neuropathologic changes associated with limbic predominant age-related TDP-43-encephalopathy^19^, and did not mention hippocampal sclerosis or evaluation of vascular pathologies. Most studies described the neuropathological hallmarks of CBD or PSP type pathology without specifying neuropathology criteria used. Many studies did not specifically evaluate early-stage^20^ PSP type pathology even in cases with Lewy bodies. These require immunostaining for phosphorylated-tau in the midbrain, globus pallidus and subthalamic nucleus, which are frequently disregarded if Lewy bodies are already detected.

Historically, clinico-pathological series were reported as a single neurodegenerative disease, rather than incorporating the multiple co-pathologies that are now increasingly recognized^21–24^. For example, one study noted that 15% of 18 pathologically defined PSP cases, had ‘marked response to levodopa’ and included 4 PSP cases with PD co-pathology (the levodopa-response in these specific 4 cases is not described)^10^. So, an apparent ‘false positive’ feature may in fact be due to PD co-pathology. The recent retrospective review of the UK Brain Bank material^17^ reported co-pathology in 37.2% individuals with PD, including DLB, MSA and AD pathology. This review reported specificity of chart clinical diagnosis (not specific criteria) at 86% for PD, but did not have further clinical details to determine how individual clinical features were impacted by the presence of co-pathologies in life. These complexities limit the binary application of individual diagnostic items and reinforce the need to interpret criteria in the broader clinical and temporal context. The likely presence of co-pathologies also challenges any estimate of diagnostic accuracy using clinical criteria, as single ‘forced choice’ diagnoses may not reflect the pathological reality in many cases.

Refining the description of clinical findings according to disease duration may be helpful. Indeed, to improve early diagnostic accuracy in the setting of clinical trials, Berg and colleagues used the 2015 MDS Clinical criteria to create “Clinically-established early PD”. These criteria removed the definition of time-frame and red flag category. Using this highly-specific definition reduced sensitivity to 68.9%^25^. Our scoping review suggests that many items may be less useful or relevant at later stages of PD, thus clarification of the descriptions or advising that some items are not applicable may be necessary to direct the physician to apply the criteria according to the disease duration of an individual patient.

In terms of practical implications of our findings for the practicing clinician; applying the current 2015 MDS Diagnostic Criteria provides good certainty of making the correct ‘clinically-probable’ PD diagnosis. Our review cautions clinicians to be careful in evaluating the degree and amplitude of ‘levodopa-response’ a patient reports, as well as subjective reports of fluctuations and dyskinesia. Adequate dopamine replacement should be ensured before excluding PD due to the presence of some ‘exclusion criteria’ or ‘red flags’, such as rapid progression of gait impairment and recurrent falls that may respond to levodopa. Criteria that were not seen in pathologically confirmed PD, and may be useful, included a normal DAT-SPECT and severe early urinary dysfunction. When applied to clinical practice, our findings illustrate that very few individual criteria rule out a specific diagnosis with absolute certainty. Moreover, neurologists with particular expertise in the field of movement disorders may be using a method of pattern recognition for diagnosis, which goes beyond any formal set of diagnostic criteria^26^. The practical implications of our findings for clinical researchers are that the current 2015 MDS Criteria are overall useful for a diagnosis of PD. However, the presence of an “absolute exclusion criteria” in an otherwise clinically-typical PD could raise the possibility of co-pathology, and thus could impact outcome for disease-modifying trials such as those targeting alpha synuclein.

In conclusion, this review of each individual item of the 2015 MDS Diagnostic criteria in pathologically proven cases of neurodegenerative parkinsonian disorders confirms that the criteria are overall reflective of a clinical diagnosis of PD but may need revision to reflect real world clinical practice. The presence of co-pathology also requires factoring in as an important potential modifier of classical clinical phenotypes and needs to be carefully documented in future clinico-pathological series. The field of movement disorders is working towards refining a Biological definition of PD^3^. This will encompass both clinical features, as well as ancillary testing including genetics, proteins, other bioassays, imaging and technology to improve specificity and sensitivity. Thus, the addition of validated biomarkers to clinical features may potentially increase specificity and practical use of the MDS Clinical criteria.

## Methods

### Search strategy

A descriptive scoping review was conducted. A medical librarian (Debbie Thomas, MLS, Becker Medical Library, Washington University School of Medicine) performed a systematic search of the literature for records including pre-defined terms for parkinsonian disorders and autopsy confirmation. Search strategies combined the use of standardized vocabulary (MeSH and Emtree) and keywords in PubMed, and Embase databases. In PubMed, date Searched: January 12, 2024 Database supplied limits: Published 1988 to the present; English language; Human studies; Exclude case reports. Number of results: 2361. Search strategy: (“autopsy proven”[tiab:~1] OR “autopsy confirmed”[tiab:~1] OR “autopsy validation”[tiab:~1] OR autopsy OR postmortem OR “pathologically confirmed”[tiab:~1]) AND (“atypical Parkinson”[tiab:~1] OR “Corticobasal Degeneration”[Mesh] OR “corticobasal syndrome”[tiab:~1] OR “Lewy Body Disease”[Mesh] OR “lewy body disease”[tiab:~1] OR “Multiple System Atrophy”[Mesh] OR “Parkinson Disease”[Mesh] OR “Parkinson Disease”/pathology OR “Parkinson disease”[tiab:~1] OR “Parkinson plus syndrome”[tiab:~1] OR “Parkinsonian Disorders”[Mesh] OR “parkinsonian syndrome”[tiab:~1] OR “Shy-Drager Syndrome”[Mesh] OR “sporadic Parkinsons”[tiab:~1] OR“Supranuclear Palsy, Progressive”[Mesh] OR “supranuclear palsy”[tiab:~1]) AND (humans[Filter]) AND (english[Filter]) NOT (animals OR rats OR rat OR mice) AND (1988:2024[pdat]) NOT (“case report*”). In Embase. Date Searched: January 12, 2024. Database supplied limits: Published 1988 to the present; English language, Human studies. Number of results: 2788. Search Strategy: (‘Parkinson disease ‘/exp/mj) OR (‘Parkinson AND disease’):ti,ab (‘Shy Drager Syndrome’/exp) OR (‘Parkinsonism’/exp) OR (parkinsonism):ti,ab OR (‘Progressive Supranuclear Palsy’/exp) OR (‘parkinson disease’):ti,ab OR (‘Diffuse Lewy Body Disease’/exp/mj) OR (‘atypical parkinson’):ti,ab OR (‘Parkinson plus syndrome’):ti,ab OR (‘multiple system atrophy’) OR (‘corticobasal syndrome’) OR (‘Parkinsonian Disorders’/exp/mj) OR (‘diffuse Lewy bodies’) AND (‘Autopsy’/exp) OR (autopsy):ti,ab OR (‘brain AND autopsy’):ti,ab OR (‘autopsy confirmed’):ti,ab OR (postmortem) OR (‘autopsy validation’:ti,ab) AND ([English]/lim AND [humans]/de AND ‘article’/it OR ‘article in press’/it OR ‘review’/it). All searches were limited to publication dates 1988 to Jan 2024, English language and human studies. A total of 5149 results were uploaded into Rayyan and 1009 duplicates were removed for a new total of 4140. PRISMA scoping review (ScR) table in supplementary material.

### Data extraction

Articles were then reviewed for clinicopathological series of PD with standard validated postmortem confirmation between 1988 and Jan 2024. The inclusion criteria required the presence of one of: clinico-pathological study of PD, autopsy-confirmed PD, pathological confirmation of PD, postmortem-confirmed PD, or neuropathological validation of PD, DLB, MSA, PSP, or CBD. The pathological diagnostic criteria used by each of the brain tissue collections in all included articles was reviewed and confirmed to be comparable across groups. (Supplementary Table 1). Cases were excluded if the clinical diagnosis was only listed as “atypical parkinsonian disorder”. Inclusion also mandated the availability of clinical information documenting at least one supportive criterion, absolute exclusion criterion, or Red Flag^7^ in at least 10 subjects. Articles were then reviewed by 2 independent movement disorder specialists serving as raters. For each paper, data was extracted on the number of patients with each of the listed 6 supportive criteria; 7 absolute exclusion criteria and 11 Red Flags. Consensus was reached by discussion between 2 reviewers. Any remaining questionable items were discussed among the group in two online meetings for clarification. For each item the percentage of patients with each pathologically confirmed diagnosis fulfilling each criterion was calculated and reported as mean (and range). Descriptive data are presented for PD, DLB, MSA, PSP and CBD for each criterion. Sensitivity and specificity of making a diagnosis of PD for each criteria were estimated (supplementary Table 2).

## Supplementary information

Supplementary Information

## Data Availability

All data generated or analysed during this study are included in this published article [and its supplementary information files].
