# Supplementary material for: Revisiting the 2015 MDS diagnostic criteria for Parkinson disease: insights from autopsy-confirmed cases
Source: NPJ Parkinsons Dis. 2025 Dec 13;11:360. doi: 10.1038/s41531-025-01206-6 (PMC12748991; doi:10.1038/s41531-025-01206-6)
Supplement: Supplementary file 1 — Supplementary Information [file 41531_2025_1206_MOESM1_ESM.pdf]

Supplementary Table 1 . Neuropathological definition according to each paper. Data was extracted from each primary paper.

Paper	Parkinson Disease	Progressive Supranuclear Palsy	Corticobasal degeneration	Multiple System Atrophy	Dementia with Lewy Bodies	Reference regarding evaluation of mixed pathologies	Additional information (extracted from paper)
Adler CH, Beach TG, Zhang N, et al. Clinical Diagnostic Accuracy of Early/Advanced Parkinson Disease: An Updated Clinicopathologic Study. Neurol Clin Pract. 2021;11(4):e414-e421.	Neuropathologic evidence of substantia nigra pigmented neuron loss and Lewy bodies.	Based on the criteria by Litvan I, Agid Y, Calne D, et al. Clinical research criteria for the diagnosis of progressive supranuclear palsy (Steele-Richardson-Olszewski syndrome): report of the NINDS-SPSP International Workshop. Neurology 1996;47:1–9	No specific mention within text.	No specific mention within text	Based on the criteria proposed by Beach TG, Adler CH, Lue L, et al. Unified staging system for Lewy body disorders: correlation with nigrostriatal degeneration, cognitive impairment and motor dysfunction. Acta Neuropathol 2009;117:613–634.	No specific mention within text	Gross and microscopic neuropathologic assessments were made by a single observer initially blinded to clinical history or clinical diagnosis.<br><br>Paraffin sections of multiple brain regions were stained with H&E and an immunohistochemical method for pathologic α-synuclein deposits using a polyclonal antibody raised against an α-synuclein peptide fragment phosphorylated at serine 129, after epitope exposure with proteinase K, to identify Lewy bodies and related neurite pathology.<br><br>Histologic evaluation of substantia nigra pigmented neuron loss was graded using H&E-stained microscopic sections.
Selikhova, M., Kempster, P. A., Revesz, T., Holton, J. L., & Lees, A. J. (2013). Neuropathological findings in benign tremulous parkinsonism. Movement Disorders, 28(2), 145-152.	Based on the criteria by Gibb WRG, Lees AJ. A comparison of clinical and pathological features of young- and old-onset Parkinson's disease. Neurology 1988; 38: 1402–1406.	No specific mention within text.	No specific mention within text.	No specific mention within text.	Based on the criteria by McKeith IG, Dickson DW, Lowe J, et al. Diagnosis and management of dementia with Lewy bodies. Third report of the DLB consortium. Neurology 2005; 65: 1863–1872.	The extent of any associated neurofibrillary tau pathology was characterized using tau immunohistochemical staining (AT8; Autogen Bioclear, Calne, UK) of standard sections. Alzheimer plaque pathology was analyzed by amyloid-β immunohistochemistry (Dako, Ely, UK) with a 4-point semiquantitative grading based on the Consortium to Establish a Registry for Alzheimer's Disease (CERAD) criteria. Four-point gradings were conducted for cerebral amyloid angiopathy and small-	Six subregions of the substantia nigra pars compacta were assessed by 2 experienced neuropathologists, blind to the clinical diagnosis. Using a consensus approach and previously published criteria,12 a score for pigmented neuronal loss (0, absent; 1, mild; 2, moderate; 3, severe) was agreed for each subregion and for the nigra as a whole. The assessment was repeated in 30% of cases to assess interrater reliability. Sections from cortical regions were stained with a monoclonal anti-α-synuclein antibody (Novocastra, Newcastle upon Tyne, UK) and assigned a Lewy-related pathology type according to the 2005 McKeith criteria. Braak PD staging was performed.<br><br>Cases in which a diagnosis of PD was not made were examined to exclude the pathological changes of multiple system atrophy, progressive supranuclear palsy, corticobasal degeneration, other tauopathies, and TDP-43 proteinopathy.

						vessel cerebrovascular pathology.	
Hughes AJ, Daniel SE, Kilford L, Lees AJ. Accuracy of clinical diagnosis of idiopathic Parkinson's disease: a clinico-pathological study of 100 cases. J Neurol Neurosurg Psychiatry. 1992 Mar;55(3):181-4. doi: 10.1136/jnnp.55.3.181.	Based on the finding of clear depletion of brainstem pigmented neurons with Lewy bodies.	Based on paper by Steele JC, Richardson JC, Olszewski J. Progressive supranuclear palsy: a heterogeneous degeneration involving the brain stem, basal ganglia and cerebellum with vertical gaze and pseudobulbar palsy, nuchal dystonia and dementia. Archives of neurology. 1964 Apr 1;10(4):333-59.	No specific mention within text.	Striatonigral involvement combined with olivopontocerebellar damaged.	No specific mention within text.	No specific mention within text.	Tissue for paraffin embedding was taken from the cortex, striatum, midbrain, pons, and medulla. Sections were stained with hematoxylin-eosin, luxol fast blue cresyl-violet, and modifeid Bielschowsky silver impregnation. On selected regions, immunohistochemistry was performed with biotin-streptavidin method and antibody to ubiquitin.
Iodice V, Lipp A, Ahlskog JE, Sandroni P, Fealey RD, Parisi JE, Matsumoto JY, Benarroch EE, Kimpinski K, Singer W, Gehrking TL, Gehrking JA, Sletten DM, Schmeichel AM, Bower JH, Gilman S, Figueroa J, Low PA. Autopsy confirmed multiple system atrophy cases: Mayo experience and role of autonomic function tests. J Neurol Neurosurg Psychiatry. 2012 Apr;83(4):453-9. doi: 10.1136/jnnp-2011-301068. Epub 2012 Jan 6..	No specific mention within text.	No specific mention within text.	No specific mention within text.	Widespread CNS α-synuclein positive glial cytoplasmic inclusions associated with neurodegenerative changes in striatonigral or olivopontocerebellar structures	No specific mention within text.	No specific mention within text.	No specific mention within text.
Litvan, I., Grimes, D.A., Lang, A.E. et al. Clinical features differentiating patients with postmortem confirmed progressive supranuclear palsy and corticobasal degeneration. J Neurol **246** (Suppl 2), II1–II5 (1999).	No specific mention within text.	Based on Collins SJ, Ahlskog JE, Parisi JE, Maraganore DM (1995) Progressive supranuclear palsy: neuropathologically based diagnostic criteria. J Neurol Neurosurg Psych 58: 167–173	No specific mention within text.	No specific mention within text.	Based on Litvan I, Grimes DA, Lang AE (in press) Phenotypes and Prognosis: Clinicopathologic Studies of Corticobasal Degeneration, Lippincott-Raven Press	No specific mention within text.	No specific mention within text.
Virmani, Tuhin, et al. "Clinicopathological characteristics of freezing of gait in autopsy-confirmed Parkinson's disease." Movement	Based on Gelb DJ, Oliver E, Gilman S. Diagnostic criteria for Parkinson	Based on Hauw JJ, Daniel SE, Dickson D, et al. Preliminary NINDS neuropathologic criteria for Steele-	No specific mention within text.	No specific mention within text.	Based on Gibb WR, Esiri MM, Lees AJ. Clinical and pathological features of diffuse cortical Lewy body disease (Lewy body	Immunosatining for ubiquitin, α-synuclein aggregates, β-amyloid, and AT8 antibodies directed against phosphorylated tau	Systematic analysis of autopsy specimens was available for 34 autopsies performed after 2003. These brains were assessed for the presence of Lewy bodies using Luxol fast blue counterstained with hematoxylin and eosin, age-related changes using

Disorders 30.14 (2015): 1874-1884.	disease. Arch Neurol 1999; **56**: 33-39. AND Forno LS. Neuropathology of Parkinson's disease. J Neuropathol Exp Neurol 1996; **55**: 259-272.	Richardson-Olszewski syndrome (progressive supranuclear palsy). Neurology 1994; **44**: 2015-2019. AND Litvan I, Hauw JJ, Bartko JJ, et al. Validity and reliability of the preliminary NINDS neuropathologic criteria for progressive supranuclear palsy and related disorders. J Neuropathol Exp Neurol 1996; **55**: 97-105.			dementia). Brain 1987; **110**: 1131-1153.	performed but regions examined not specified. .	Bielschowsky silver stain, in addition to antibodies directed against ubiquitin, α-synuclein aggregates, β-amyloid, and AT8 antibodies directed against phosphorylated tau. Specimens from autopsies performed before 2003 were more heterogeneous, but all 18 were assessed for Lewy bodies by hematoxylin-eosin and a subset for ubiquitin (n = 7), α-synuclein (n = 5), and thioflavin-S (n = 17).
Rajput AH, Voll A, Rajput ML, Robinson CA, Rajput A. Course in Parkinson disease subtypes: A 39-year clinicopathologic study. Neurology. 2009 Jul 21;73(3):206-12..	Marked substantia nigra (SN) neuronal loss and Lewy body (LB) inclusions	No specific mention within text.	No specific mention within text.	No specific mention within text.	No specific mention within text.	Concomitant pathologic findings which resulted in exclusion included progressive supranuclear palsy (PSP), corticobasal ganglia degeneration (CBG), multiple system atrophy (MSA), ablative surgery, and basal ganglia stroke.	Pathologic studies were performed by 9 Canadian board-certified neuropathologists. The pathologist was aware of history of parkinsonism. All the available informative staining techniques, including silver stains and as they became commercially available, ubiquitin, tau, and α-synuclein immunostains, were used routinely. Complete neuropathology report was issued on each case. Only those patients who had a marked substantia nigra (SN) neuronal loss and Lewy body (LB) inclusions without evidence of other Parkinson-related pathology were included.
Ishida C, Takahashi K, Kato-Motozaki Y, Tagami A, Komai K. Effectiveness of Levodopa in Patients with Multiple System Atrophy and Associated Clinicopathological Features. Intern Med. 2021 Feb 1;60(3):367-372.	No specific mention within text.	No specific mention within text.	No specific mention within text.	Based on neurodegenerative findings in the substantia nigra-striatal system and the olivopontocerebellar system, a large number of α-synuclein-positive glial cytoplasmic inclusions, characteristic of MSA.	No specific mention within text.	No specific mention within text.	The microscopic findings of the putamen were observed in three coronal planes: the mammillary body, anterior commissure, and nucleus accumbens. The putaminal neurodegeneration of each plane was then qualitatively classified into 4 levels as either “almost normal”, “mild”, “moderate”, or “severe”. Datasets were further graded from G0-G3 as follows: G0, “almost normal” in all three planes; G1, “mild” to “moderate” in the mammillary body and anterior commissure planes, and “almost normal” in the nucleus accumbens plane; G2, “moderate” to “severe” in the

							mammillary body and anterior commissure planes, and “mild” in the nucleus accumbens plane; and G3, “moderate” to “severe” in all three planes.
Müller, Jörg, et al. "Freezing of gait in postmortem-confirmed atypical parkinsonism." Movement Disorders: Official Journal of the Movement Disorder Society 17.5 (2002): 1041-1045.	No specific mention within text.	Based on Litvan I, Agid Y, Calne D, et al. Clinical research criteria for the diagnosis of progressive supranuclear palsy (Steele-Richardson-Olszewski syndrome): report of the NINDS-SPSP international workshop. Neurology 1996; **47**: 1–9.	No specific mention within text.	No specific mention within text.	McKeith IG, Galasko D, Kosaka K, et al. Consensus guidelines for the clinical and pathological diagnosis of dementia with Lewy bodies (DLB): report of the consortium on DLB international workshop. Neurology 1996 ; **47**: 1113–1124.	No specific mention within text.	The presence or absence of cell loss and gliosis of selected regions potentially involved in the pathophysiology of FoG was retrospectively abstracted from the neuropathological files and the following sites were included: frontal cortex, putamen, caudate nucleus, globus pallidus, substantia nigra, locus coeruleus and pontine nuclei.
Silveira-Moriyama L, Hughes G, Church A, Ayling H, Williams DR, Petrie A, Holton J, Revesz T, Kingsbury A, Morris HR, Burn DJ. Hyposmia in progressive supranuclear palsy. Movement Disorders. 2010 Apr 15;25(5):570-7.	No specific mention within text.	Based on Litvan I,Hauw JJ,Bartko JJ, et al. Validity and reliability of the preliminary NINDS neuropathologic criteria for progressive supranuclear palsy and related disorders. J Neuropathol Exp Neurol 1996; **55**: 97–105.	No specific mention within text.	No specific mention within text.	McKeith IG, Galasko D, Kosaka K, et al. Consensus guidelines for the clinical and pathological diagnosis of dementia with Lewy bodies (DLB): report of the consortium on DLB international workshop. Neurology 1996 ; **47**: 1113–1124.	Analysis of neuritic plaque density estimated according to The Consortium to Establish a Registry for Alzheimer's Disease (CERAD) criteria, Braak and Braak staging of neurofibrillary tangle pathology determined using tau immunohistochemistr y,	Neuropathological data were obtained including analysis of α-synuclein Braak staging according to screening of regions of vulnerability using α-synuclein immunohistochemistry; cases in which no α-synuclein related pathology was found in any of the areas screened received a Braak stage score of “zero.”<br><br>**Evaluation of the Rhinencephalon**<br>Sections of 8 and 13 µm from formalin-fixed wax-embedded blocks of the mesial temporal cortex and forebrain were cut in all subjects, but the olfactory tract was only available for examination in two. The 13 µm sections were stained with Luxol fast blue/cresyl violet for the delimitation of the areas of interest using established anatomical guidelines, which included the olfactory tract, anterior olfactory nucleus, frontal and temporal segments of the piriform cortex, and anterior cortical nucleus of the amygdala and periamygdaloid cortex. Adjacent 8 µm sections were used for immunohistochemistry with a monoclonal antibody to α-syn (Novocastra, Newcastle upon Tyne, UK; 1:75), tau (AT8 antibody, Thermo Scientific, Loughborough, UK; 1:200), and the 3R (RD3) and 4R (RD4) tau-specific monoclonal antibodies(Upstate/Millipore, Dundee, UK; RD3, 1:3,000; RD4, 1:100). The severity of

							Lewy body and Lewy neurite pathology was graded in each of the areas separately following the criteria proposed by McKeith et al. for semiquantitative grading of lesion density in a zero to four scale as previously described. Severity of tau-related pathology was graded according to procedures that are routine.
Miki Y, Tsushima E, Foti SC, Strand KM, Asi YT, Yamamoto AK, Bettencourt C, Oliveira MCB, De Pablo-Fernández E, Jaunmuktane Z, Lees AJ, Wakabayashi K, Warner TT, Quinn N, Holton JL, Ling H. Identification of multiple system atrophy mimicking Parkinson's disease or progressive supranuclear palsy. Brain. 2021 May 7;144(4):1138-1151.	Based on McKeith IG, Boeve BF, Dickson DW, et al. Diagnosis and management of dementia with Lewy bodies: Fourth consensus report of the DLB Consortium. Neurology. 2017;89:88–100.	No specific mention within text.	No specific mention within text.	Based on Ozawa T, Paviour D, Quinn NP, et al. The spectrum of pathological involvement of the striatonigral and olivopontocerebellar systems in multiple system atrophy: Clinicopathological correlations. Brain. 2004;127:2657–2671	Based on McKeith IG, Boeve BF, Dickson DW, et al. Diagnosis and management of dementia with Lewy bodies: Fourth consensus report of the DLB Consortium. Neurology. 2017;89:88–100.	The Bielschowsky silver impregnation was performed in some cases for the assessments of neuritic plaques and neurofibrillary tangles. Neuritic plaques and neurofibrillary tangles were evaluated according to the Consortium to Establish a Registry for Alzheimer's Disease (CERAD) scheme and Braak and Braak neurofibrillary tangle stage, respectively. Amyloid-β deposits are first encountered in the basal portions of the frontal, temporal and occipital lobes, and then spread to other regions.If the parietal lobe was not available for evaluating neuritic plaques, we evaluated neuritic plaques using the frontal and temporal lobes that are considered to have more amyloid deposits than the parietal lobe.	The brains were fixed in 10% buffered formalin for 3 weeks. Immunohistochemical analysis was performed using 8-µm thick, formalin-fixed, paraffin-embedded sections from multiple brain regions. The sections were subjected to immunohistochemical processing using the avidin-biotin-peroxidase complex method with diaminobenzidine as the chromogen. The primary antibodies used were anti-amyloid-β (M0872; Dako; 1:100), anti-α-synuclein (MA1-90342; Thermo Scientific; 1:1500) and anti-phosphorylated tau (MN1020; Thermo Scientific; 1:600) antibodies. The Bielschowsky silver impregnation was performed in some cases for the assessments of neuritic plaques and neurofibrillary tangles. MSA was classified into four pathological subtypes: MSA, striatonigral degeneration predominant type (MSA-SND); MSA, olivopontocerebellar predominant type (MSA-OPCA); MSA with equal involvement of SND and OPCA (MSA-SND=OPCA); and MSA with minimal change based on previously published criteria. Lewy pathology [brainstem-predominant, limbic (transitional) and diffuse neocortical] was assigned based upon the pattern of Lewy body-related pathology according to the consensus criteria for pathological assessment of dementia with Lewy bodies. We used sections stained with haematoxylin and eosin to assess concomitant Lewy bodies in the substantia nigra and the locus coeruleus due to difficulties in discriminating concomitant Lewy bodies in MSA from neuronal cytoplasmic inclusions, another characteristic feature of MSA, with the use of α-synuclein immunohistochemistry.

Yasuo Miki, Sandrine C Foti, Yasmine T Asi, Eiki Tsushima, Niall Quinn, Helen Ling, Janice L Holton, Improving diagnostic accuracy of multiple system atrophy: a clinicopathological study, Brain, Volume 142, Issue 9, September 2019, Pages 2813–2827	Based on McKeith IG, Boeve BF, Dickson DW, et al. Diagnosis and management of dementia with Lewy bodies: Fourth consensus report of the DLB Consortium. Neurology. 2017;89:88–100.	No specific mention within text.	No specific mention within text.	Based on Ozawa T, Paviour D, Quinn NP, et al. The spectrum of pathological involvement of the striatonigral and olivopontocerebellar systems in multiple system atrophy: Clinicopathological correlations. Brain. 2004;127:2657–2671	Based on McKeith IG, Boeve BF, Dickson DW, et al. Diagnosis and management of dementia with Lewy bodies: Fourth consensus report of the DLB Consortium. Neurology. 2017;89:88–100.	Neuritic plaques and neurofibrillary tangles were also evaluated according to the Consortium to Establish a Registry for Alzheimer's Disease (CERAD) scheme and Braak NFT stage, respectively	The brains were fixed with 10% buffered formalin for 3 weeks. Formalin-fixed, paraffin-embedded sections (8-µm thick) were cut from multiple regions throughout the brain. Sections were first stained with haematoxylin and eosin. They were then subjected to immunohistochemical processing with the avidin-biotin-peroxidase complex method with diaminobenzidine as the chromogen. For routine histological investigations, mouse monoclonal antibodies against amyloid-β (M0872; Dako; 1:100), α-synuclein (MA1-90342; Thermo Scientific; 1:1500), tau (MN1020; Thermo Scientific; 1:600) and transactivation response DNA-binding protein 43 kDa (TDP-43) (H00023435-M01; Abnova; 1:6000) and a rabbit polyclonal antibody against ubiquitin (Z0458; Dako; 1:200) were used. Sections in some cases were also stained with the Bielschowsky silver impregnation for the assessments of neuritic plaques and neurofibrillary tangles. Based on the degree of neuronal cell loss and glial cytoplasmic inclusions, MSA was subclassified into MSA of striatonigral degeneration predominant type (MSA-SND), MSA of olivopontocerebellar predominant type (MSA-OPCA) and MSA with equal involvement of SND and OPCA (MSA-SND=OPCA). Lewy body type (brainstem predominant, limbic and diffuse neocortical) was assigned based on pattern of Lewy-related pathology according to the consensus criteria for pathological assessment of dementia with Lewy bodies.
Martin WRW, Miles M, Zhong Q, Hartlein J, Racette BA, Norris SA, Ushe M, Maiti B, Criswell S, Davis AA, Kotzbauer PT, Cairns NJ, Perrin RJ, Perlmutter JS. Is Levodopa Response a Valid Indicator of Parkinson's Disease? Mov Disord. 2021 Apr;36(4):948-954.	Based on Braak H, Del Tredici K, Rüb U, de Vos RA, Jansen Steur EN, Braak E. Staging of brain pathology related to sporadic Parkinson's disease. Neurobiol Aging 2003; 24: 197–211.	Based on Litvan I, Hauw JJ, Bartko JJ, et al. Validity and reliability of the preliminary NINDS neuropathologic criteria for progressive supranuclear palsy and related disorders. J Neuropathol Exp Neurol 1996; 55: 97–105	Based on Dickson DW, Bergeron C, Chin SS, et al. Office of Rare Diseases neuropathologic criteria for corticobasal degeneration. J Neuropath Exp Neurol 2002; 61: 935–946.	Based on Gilman S, Low PA, Quinn N, et al. Consensus statement on the diagnosis of multiple system atrophy. J Neurol Sci 1999; 163: 94–98	Based on McKeith IG, Galasko D, Kosaka K, et al. Consensus guidelines for the clinical and pathologic diagnosis of dementia with Lewy bodies (DLB): report of the Consortium on DLB International Workshop. Neurology 1996; 47: 1113–1124	Cases with multiple pathologies were identified, looking specifically for PD, PSP, CBD, and MSA as well as a high likelihood of AD based on NIA-Reagan diagnostic criteria, nonvascular amyloid plaque deposits not meeting these criteria for an AD diagnosis, TDP43 neuronal immunoreactivity, and	Immunohistochemistry (IHC) for alpha-synuclein using the LB509 monoclonal antibody was introduced in 1998, and was supplanted by IHC using phosphorylation-specific anti-alpha-synuclein antibodies in 2009.

						primary age-related tauopathy (PART). Those with multiple pathologies were arbitrarily classified for further analysis by the non-PD movement disorder pathology.	
Wenning GK, Tison F, Ben Shlomo Y, Daniel SE, Quinn NP. Multiple system atrophy: a review of 203 pathologically proven cases. Movement disorders. 1997 Mar;12(2):133-47.	No specific mention within text.	No specific mention within text.	No specific mention within text.	Based on Quinn N. Multiple system atrophy. In: Marsden CD, FahnS, eds. Movement disorders, 111. London: Butterworth-Heine-mann, 1994:262-281 AND Papp MI, Kahn JE, Lantos PL. Glial cytoplasmic inclusionsin the CNS in patients with multiple system atrophy (striatoni-gral degeneration, olivopontocerebellar atrophy and Shy-Drager syndrome). J Neurol Sci 1989;94:79-100	No specific mention within text.	No specific mention within text.	The degree of cell loss and gliosis was usually re-ported as absent, mild, moderate, or severe. These assessments were retrospectively converted to a 4-point scale ranging from normal (0 score) to severe (score of 3). The following sites were included: putamen, caudate nucleus, globus pallidus, thalamus, sub-thalamic nucleus, substantia nigra, locus ceruleus,dorsal vagal nucleus, vestibular nuclei, nucleus ambi-guus, pontine nuclei, inferior olives, cerebellar Purkinje cells, dentate nucleus, intermediolateral col-umn, anterior horn cells, Onuf's nucleus, andpyramidal tracts. Severity counts for substantia nigra,putamen, caudate nuclei, and globus pallidus were averaged as an SND score (range, 0-3); severity counts in the inferior olives, pontine nuclei, and cerebellar Purkinje cells were averaged as an OPCA score(range, 0-3); and (when available) severity counts inintermediolateral cell columns, anterior horn cells, pyramidal tracts, and Onuf 's nucleus were averaged as a spinal cord score (range, 0-3)
Figueroa JJ, Singer W, Parsaik A, Benarroch EE, Ahlskog JE, Fealey RD, Parisi JE, Sandroni P, Mandrekar J, Iodice V, Low PA, Bower JH. Multiple system atrophy: prognostic indicators of survival. Mov Disord. 2014 Aug;29(9):1151-7.	No specific mention within text.	No specific mention within text.	No specific mention within text.	Based on Gilman S, Wenning GK, Low PA, Brooks DJ, Mathias CJ, Trojanowski JQ, Wood NW, Colosimo C, Durr A, Fowler CJ, et al. Second consensus statement on the diagnosis of multiple system atrophy. Neurology. 2008;71:670–676..	No specific mention within text.	No specific mention within text.	No specific mention within text.
Geut H, Hepp DH, Foncke E, Berendse HW, Rozemuller JM, Huitinga I, van de Berg WDJ. Neuropathological correlates of parkinsonian disorders in a large Dutch	Based on Dickson DW, Braak H, Duda JE, Duyckaerts C, Gasser T, Halliday GM, Hardy J, Leverenz JB, Del	Based on Litvan I, Agid Y, Calne D, Campbell G, Dubois B, Duvoisin RC, Goetz CG, Golbe LI, Grafman J, Growdon JH, et al. Clinical	Mackenzie IR, Neumann M, Bigio EH, Cairns NJ, Alafuzoff I, Kril J, Kovacs GG, Ghetti B, Halliday G, Holm IE, et al. Nomenclature and	Based on Gilman S, Wenning GK, Low PA, Brooks DJ, Mathias CJ, Trojanowski JQ, Wood NW, Colosimo C, Durr A, Fowler CJ, et al. Second consensus statement on the diagnosis	No specific mention within text.	Thal amyloid-β phase, Braak stage for neurofibrillary (NFT) pathology and CERAD neuritic plaque scores were determined according to the most	Autopsy was performed using a standardized protocol by the NBB (open access: www.brainbank.nl). Post-mortem examination was performed by two experienced neuropathologists (JR and WK). LP was defined as the presence of Lewy bodies and Lewy neurites. Donors

autopsy series. Acta Neuropathol Commun. 2020 Mar 26;8(1):39.	Tredici K, Wszolek ZK, et al. Neuropathological assessment of Parkinson's disease: refining the diagnostic criteria. Lancet Neurol. 2009;8:1150–1157.	research criteria for the diagnosis of progressive supranuclear palsy (Steele-Richardson-Olszewski syndrome): report of the NINDS-SPSP international workshop. Neurology. 1996;47:1–9.	nosology for neuropathologic subtypes of frontotemporal lobar degeneration: an update. Acta Neuropathol. 2010;119:1–4.	of multiple system atrophy. Neurology. 2008;71:670–676. doi: 10.1212/01.wnl.0000324625.00404.15.		recent National Institute on Aging – Alzheimer's Association guidelines, and Braak and McKeith stages for LP were determined according to the BrainNet Europe guidelines.	were diagnosed with vascular parkinsonism when either vascular lesions were present in nigrostriatal regions, or features of small vessel disease, such as enlargement of perivascular spaces, perivascular pallor, gliosis or hyaline thickening of the vascular walls, were present in periventricular regions, in the absence of other neuropathological lesions apart from age-related changes.
Martin WRW, Hartlein J, Racette BA, Cairns N, Perlmutter JS. Pathologic correlates of supranuclear gaze palsy with parkinsonism. Parkinsonism Relat Disord. 2017 May;38:68-71.	Based on Braak H, Del Tredici K, Rüb U, de Vos RA, Jansen Steur EN, Braak E. Staging of brain pathology related to sporadic Parkinson's disease. Neurobiol Aging 2003; 24: 197–211.	Based on Litvan I, Hauw JJ, Bartko JJ, et al. Validity and reliability of the preliminary NINDS neuropathologic criteria for progressive supranuclear palsy and related disorders. J Neuropathol Exp Neurol 1996; 55: 97–105	Based on Dickson DW, Bergeron C, Chin SS, et al. Office of Rare Diseases neuropathologic criteria for corticobasal degeneration. J Neuropath Exp Neurol 2002; 61: 935–946.	Based on Gilman S, Low PA, Quinn N, et al. Consensus statement on the diagnosis of multiple system atrophy. J Neurol Sci 1999; 163: 94–98	Based on McKeith IG, Galasko D, Kosaka K, et al. Consensus guidelines for the clinical and pathologic diagnosis of dementia with Lewy bodies (DLB): report of the Consortium on DLB International Workshop. Neurology 1996; 47: 1113–1124	No specific mention within text.	The diagnosis of Parkinson disease (PD) was based on the loss of pigmented neurons from the substantia nigra combined with the presence of Lewy bodies. Cortical alpha-synuclein-positive inclusions consistent with Lewy bodies, although not required for the diagnosis of PD, were present in many of these patients. PSP patients had tau-positive neurofibrillary tangles in the typical distribution in cortex and subcortical nuclei.[5] Those with multiple system atrophy (MSA) had α-synuclein–positive glial cytoplasmic inclusions with neurodegenerative changes in striatonigral or olivopontocerebellar structures.<br><br>The diagnosis of corticobasal ganglia degeneration (CBD) was based on the presence of tau-positive neuronal inclusions, astrocytic plaques and neuronal loss in cortex and basal ganglia.
De Pablo-Fernández E, Lees AJ, Holton JL, Warner TT. Prognosis and Neuropathologic Correlation of Clinical Subtypes of Parkinson Disease. JAMA Neurol. 2019 Apr 1;76(4):470-479.	Based on Braak H, Del Tredici K, Rüb U, de Vos RA, Jansen Steur EN, Braak E. Staging of brain pathology related to sporadic Parkinson's disease. Neurobiol Aging. 2003;24(2):197-211. AND Alafuzoff I, Ince PG, Arzberger T, et al. Staging/typing of Lewy body related alpha-synuclein	No specific mention within text.	No specific mention within text.	No specific mention within text.	Based on McKeith IG, Boeve BF, Dickson DW, et al. Diagnosis and management of dementia with Lewy bodies: fourth consensus report of the DLB Consortium. Neurology. 2017;89(1):88-100.	Severity of neurofibrillary tangle pathology was assessed using specific immunohistochemistry against hyperphosphorylated tau protein and the staging system described by Braak and Braak.<br><br>Amyloid β immunostaining was used for the assessment of amyloid β deposition, and	Formalin-fixed brain tissue samples were examined with immunohistochemistry using standard protocols. Representative sections of brainstem, basal forebrain, and neocortical areas were graded for distribution and severity of Lewy pathology, and each individual was assigned to a Lewy body subtype (brainstem, limbic, or diffuse neocortical) and a Braak stage (stage 1-6) based on Lewy body scores.

	pathology: a study of the BrainNet Europe Consortium. Acta Neuropathol. 2009;117(6):635-652.					neuritic plaques and their severity and extent were graded based on the classifications proposed by Thal et al and the Consortium to Establish a Registry for Alzheimer's Disease protocol.<br><br>Global Alzheimer disease neuropathologic changes were assessed using the ABC scoring system proposed by the National Institute on Aging–Alzheimer's Association guidelines (absent, low, intermediate, and high) based on the combination of neurofibrillary tangle, amyloid β deposition, and neuritic plaque severity.	
Rajput AH, Pahwa R, Pahwa P, Rajput A. Prognostic significance of the onset mode in parkinsonism. Neurology. 1993 Apr;43(4):829-.	No specific mention within text.	No specific mention within text.	No specific mention within text.	No specific mention within text.	No specific mention within text.	No specific mention within text.	No specific mention within text.
Wenning GK, Ebersbach G, Verny M, Chaudhuri KR, Jellinger K, McKee A, Poewe W, Litvan I. Progression of falls in postmortem-confirmed parkinsonian disorders. Movement disorders: official journal of the Movement Disorder Society. 1999 Nov;14(6):947-50.	No specific mention within text.	Based on Litvan I, Hauw JJ, Bartko JJ, et al. Validity and reliability of the preliminary NINDS neuropathologic criteria for progressive supranuclear palsy and related disorders. J Neuropathol Exp Neurol 1996; 55: 97–105	No specific mention within text.	No specific mention within text.	Based on Kosaka K. Diffuse Lewy body disease in Japan. J Neurol 1990; 237:197–204	No specific mention within text.	The cases met the neuropathologic NINDS criteria for the diagnosis of PSP and related disorders and Kosaka's criteria for Lewy body disease.

Colosimo C, Albanese A, Hughes AJ, de Bruin VM, Lees AJ. Some specific clinical features differentiate multiple system atrophy (striatonigral variety) from Parkinson's disease. Arch Neurol. 1995 Mar;52(3):294-8.	No specific mention within text.	No specific mention within text.	No specific mention within text.	Based on Fearnley JM, Lees AJ. Striatonigral degeneration: a clinicopathological study. Brain. 1990;113:1823-1842.	No specific mention within text.	No specific mention within text.	Twenty-seven cases of pathologically proven multiple system atrophy (MSA) were collected from 1984 to 1992 by the Parkinson's Disease Society Brain Tissue Bank in London, England. This facility receives donor tissue from parkinsonian patients, most of whom had been examined at annual intervals by a panel of experienced neurologists.<br><br>These 27 MSA cases accounted for 13% of the 208 consecutive brains collected during the eight-year period, making MSA the second most common pathological diagnosis after Parkinson's disease (PD). Half-brains fixed in 10% neutral formalin were available for examination using standard neuropathological methods.<br><br>The diagnosis of MSA was made according to already published neuropathological criteria. All cases showed striatonigral involvement combined (in most instances) with some degree of olivopontocerebellar damage.
Hughes AJ, Colosimo C, Kleedorfer B, Daniel SE, Lees AJ. The dopaminergic response in multiple system atrophy. J Neurol Neurosurg Psychiatry. 1992;55(11):1009-1013.	No specific mention within text.	No specific mention within text.	No specific mention within text.	Based on Fearnley JM, Lees AJ. Striatonigral degeneration: a clinicopathological study. Brain. 1990;113:1823-1842.	No specific mention within text.	No specific mention within text.	Half-brains fixed in 10% neutral formalin were available for examination using standard neuropathological methods.
Wenning GK, Scherfler C, Granata R, Bösch S, Verny M, Chaudhuri KR, Jellinger K, Poewe W, Litvan I. Time course of symptomatic orthostatic hypotension and urinary incontinence in patients with postmortem confirmed parkinsonian syndromes: a clinicopathological study. J Neurol Neurosurg Psychiatry. 1999 Nov;67(5):620-3	No specific mention within text.	Based on Litvan I, Hauw JJ, Bartko JJ, et al. Validity and reliability of the preliminary NINDS neuropathologic criteria for progressive supranuclear palsy and related disorders. J Neuropathol Exp Neurol 1996; 55: 97–105	No specific mention within text.	No specific mention within text.	Based on Kosaka K. Diffuse Lewy body disease in Japan. J Neurol 1990; 237:197–204	No specific mention within text.	No specific mention within text.
Jellinger KA. Very old onset parkinsonism: A clinical-pathological study.	Based on S.E. Daniel, A.J. Lees, Parkinson's disease society	Based on Litvan I, Hauw JJ, Bartko JJ, et al. Validity and reliability of the	Based on Litvan I, Hauw JJ, Bartko JJ, et al. Validity and reliability of the	Based on Gilman S, Wenning GK, Low PA, Brooks DJ, Mathias CJ, Trojanowski JQ, Wood NW,	Based on I.G. McKeith, D. Galasko, K. Kosaka, E.K. Perry, D.W. Dickson, L.A. Hansen, D.P. Salmon, J.	Description of combination of pathologies.	Neuropathological studies were performed according to established protocols using silver impregnation, Gallyas-Braak stain and immunohistochemistry for α-synuclein,

Parkinsonism Relat Disord. 2018 Dec;57:39-43..	brain bank, London: overview and research, J. Neural. Transm. Suppl. 39 (1993) 165–172.	preliminary NINDS neuropathologic criteria for progressive supranuclear palsy and related disorders. J Neuropathol Exp Neurol 1996; 55: 97–105	preliminary NINDS neuropathologic criteria for progressive supranuclear palsy and related disorders. J Neuropathol Exp Neurol 1996; 55: 97–105	Colosimo C, Durr A, Fowler CJ, et al. Second consensus statement on the diagnosis of multiple system atrophy. Neurology. 2008;71:670–676.	Lowe, S.S. Mirra, E.J. Byrne, G. Lennox, N.P. Quinn, J.A. Edwardson, P.G. Ince, C. Bergeron, A. Burns, B.L. Miller, S. Lovestone, D. Collerton, E.N. Jansen, C. Ballard, R.A. de Vos, G.K. Wilcock, K.A. Jellinger, R.H. Perry, Consensus guidelines for the clinical and pathologic diagnosis of de- mentia with Lewy bodies (DLB): report of the consortium on DLB international workshop, Neurology 47 (1996) 1113–1124.		β-amyloid and tau. Neuropathological diagnosis was performed unaware of clinical information and finally, was compared with the clinical data
Wenning GK, Ben-Shlomo Y, Hughes A, Daniel SE, Lees A, Quinn NP. What clinical features are most useful to distinguish definite multiple system atrophy from Parkinson's disease? J Neurol Neurosurg Psychiatry. 2000 Apr;68(4):434-40.;	Depletion of pigmented neurons in the substantia nigra and locus ceruleus, with Lewy bodies in some remaining nerve cells and else- where in the nervous system. The striatum was normal and there were no glial cytoplasmic inclusions or additional pathology to account for the presence of parkinsonism.<br><br>Hughes AJ, Daniel SE, Kilford L, et al. Accuracy of clinical diagnosis of idiopathic Parkinson's disease: a clinicopatho- logical study of 100 cases. J Neurol Neurosurg	No specific mention within text.	No specific mention within text.	Neuronal cell loss and gliosis were observed in a selection of at least two of the following structures: striatum, substantia nigra, locus ceruleus, pontine nuclei and middle cerebellar peduncles, cerebellar Purkinje cells, inferior olives, and dorsal vagal nuclei. In addition, characteristic glial cytoplasmic argentophilic inclusions were present in the primary or secondary motor areas and basal ganglia.<br><br>Because the striatal lesion in MSA may appear subtle or even absent using conventional histological methods, immunohistochemistry for glial fibrillary acidic protein (GFAP) was performed in all patients, in addition to routine stains such as cresyl violet (Nissl) and Luxol fast blue.	No specific mention within text.	No specific mention within text.	No specific mention within text.

	Psychiatry 1992a;**55**:181–4.			Based on Gray F, Vincent D, Hauw JJ. Quantitative study of lateral horn cells in 15 cases of multiple system atrophy. Acta Neu- ropathol 1988;75:513–18. AND Quinn N. Multiple system atrophy. In: Marsden CD, Fahn S, eds. Movement disorders 3. London: Butterworth- Heinemann, 1994:262–81.			
Hughes AJ, Ben-Shlomo Y, Daniel SE, Lees AJ. What features improve the accuracy of clinical diagnosis in Parkinson's disease: a clinicopathologic study. Neurology. 1992 Jun;42(6):1142-.	The diagnosis of Parkinson's disease (PD) was based on the identification of clear depletion of pigmented neurons in the substantia nigra, with Lewy bodies present in some of the remaining nerve cells.[8] In all cases where Lewy bodies were difficult to detect, at least three hematoxylin-eosin–stained 7-µm sections of the midbrain were examined. In cases lacking the pathological changes characteristic of PD, alternative diagnoses were established using accepted neuropathological criteria	Based on Steele JC, Richardson JC, Olszewski J. Progressive supranuclear palsy. Arch Neurol 1964;10:333-359	No specific mention within text.	No specific mention within text.	No specific mention within text.	No specific mention within text.	Half-brains fixed in 10% neutral formalin were examined using standard neuropathologic method.
Litvan I, Goetz CG, Jankovic J, Wenning GK, Booth V, Bartko JJ, McKee A, Jellinger K, Lai EC, Brandel JP, Verny M, Chaudhuri KR, Pearce RK, Agid Y. What is the accuracy of the clinical	Based on Hughes A.J., Daniel S.E., Kilford L., Lees A.J. Accuracy of clinical diagnosis of idiopathic Parkinson's	Based on Litvan I., Agid Y., Jankovic J., et al. Accuracy of clinical criteria for the diagnosis of progressive supranuclear palsy	Based on Litvan I., Agid Y., Goetz C., et al. Accuracy of the clinical diagnosis of corticobasal degeneration: a clinicopathological	Based on Wenning G.K., Tison F., Ben-Shlomo Y., Daniel S.E., Quinn N.P. Multiple system atrophy: a review of 203 pathologically proven cases. Mov Disord.	Based on McKeith I.G., Galasko D., Kosaka K., et al. Consensus guidelines for the clinical and pathological diagnosis of dementia with Lewy bodies (DLB): report of the	No specific mention within text.	No specific mention within text.

diagnosis of multiple system atrophy? A clinicopathologic study. Arch Neurol. 1997 Aug;54(8):937-44.	disease: a clinico-pathological study of 100 cases. J Neurol Neurosurg Psychiatry. 1992;55:181–184.	(Steele-Richardson-Olszewski syndrome). Neurology. 1996;46:922–930.And Hauw J.J., Daniel S.E., Dickson D., et al. Preliminary NINDS neuropathologic criteria for Steele-Richardson-Olszewski syndrome (progressive supranuclear palsy). Neurology. 1994;44:2015–2019.	study. Neurology. 1997;48:119–125.	1997;12:133–147.And Lantos P.L. Neuropathological diagnostic criteria of multiple system atrophy: a review. In: Cruz-Sanchez F.F., Ravid R., Cuzner M.L., eds. Neuropathological Diagnostic Criteria for Brain Banking. Amsterdam, the Netherlands: IOS Press; 1995:116–121.	Consortium on DLB International Workshop. Neurology. 1996;47:1113–1124.		
Koga S, Aoki N, Uitti RJ, et al. When DLB, PD, and PSP masquerade as MSA: an autopsy study of 134 patients. Neurology. 2015;85(5):404-412.	Based on Dickson DW, Braak H, Duda JE, et al. Neuropathological assessment of Parkinson's disease: refining the diagnostic criteria. Lancet Neurol 2009;8:1150–1157	Based on Litvan I, Agid Y, Calne D, et al. Clinical research criteria for the diagnosis of progressive supranuclear palsy (Steele-Richardson-Olszewski syndrome): report of the NINDS-SPSP international workshop. Neurology 1996;47:1–9.	Based on Litvan I, Agid Y, Calne D, et al. Clinical research criteria for the diagnosis of progressive supranuclear palsy (Steele-Richardson-Olszewski syndrome): report of the NINDS-SPSP international workshop. Neurology 1996;47:1–9.	Based on Trojanowski JQ, Revesz T. Proposed neuropathological criteria for the post mortem diagnosis of multiple system atrophy. Neuropathol Appl Neurobiol 2007;33:615–62	Based on McKeith IG, Dickson DW, Lowe J, et al. Diagnosis and management of dementia with Lewy bodies: third report of the DLB Consortium. Neurology 2005;65:1863–1872	All cases underwent a standardized neuropathologic assessment for Alzheimer-type pathology.	All cases underwent a standardized neuropathologic assessment for Alzheimer-type and Lewy-related pathologies as previously reported.[9]Braak neurofibrillary tangle (NFT) stageand Thal amyloid phase were assigned to each case based upon thioflavin S fluorescent microscopy. Immunohistochemistry for α-synuclein (NACP; 1:3,000) was used to establish neuropathologic diagnosis of MSA. MSA was subclassified as MSA with predominantly striatonigral involvement (MSA-SND), MSA with predominantly olivopontocerebellar involvement (MSA-OPCA), and MSA with equally severe involvement of striatonigral and olivopontocerebellar systems (MSA-SND/OPCA). Lewy-related pathology was assessed in cortex, amygdala, basal forebrain, and brainstem, and classified as brainstem, transitional, or diffuse Lewy body disease. Lewy body subtype and degree of Alzheimer-type pathology were used to classify cases as low, intermediate, or high likelihood of dementia with Lewy bodies (DLB) according to the Third Consortium on Dementia with Lewy Bodies (CDLB) recommendations; a pathologic diagnosis of DLB was assigned to cases with intermediate or high likelihood of CDLB. A pathologic diagnosis of Parkinson disease (PD) required moderate to severe neuronal loss in the substantia nigra and CDLB scores of low likelihood.

Supplementary Table 2 Sensitivity and specificity estimates of individual criteria of the 2015 MDS Diagnostic Criteria for the diagnosis of PD

Supportive Criteria	Sensitivity	Specificity
Clear and dramatic beneficial response to dopaminergic therapy. During initial treatment patient returned to normal or near normal.	84.1 (69-100)	66.8 (45– 88)
Marked improvement with dose increases or marked worsening with dose decreases -Or unequivocal and marked on/off with predictable end-of-dose wearing off. -	57.1 (33 – 84)	68.4(60 – 75)
Presence of levodopa-induced dyskinesia(s)	61.7 (55– 81)	63.4(56 – 73)
Rest tremor of a limb	68 (43 -91)	73.2 (62-89)
Presence of olfactory loss	95	ND
Presence of Cardiosympathetic denervation (MIBG SPECT)	ND	ND

Absolute Exclusion Criteria	Sensitivity	Specificity
Cerebellar /abnormalities/ Symptoms		
Gait Ataxia	ND	32.9 (11- 48)
Limb Ataxia	ND	35.1 (5 – 46)
Non-specific Ataxia	99 (98-100)	49.4 (32 – 71)
Downward Supranuclear gaze palsy or selective slowing of downward vertical saccades	93	53.3 (20 – 75)
Frontal behavior dementia / frontal dementia/progressive aphasia	ND	13.5 (7 – 25)
Parkinsonian features restricted to lower limbs for more than 3 years	ND	ND
Absence of observable response to high dose levodopa despite at least moderate severity of disease	49 (49 -50)	44.5 (27 -75)
Unequivocal Cortical sensory loss; limb apraxia; progressive aphasia	ND	ND
FP CIT-SPECT and normal/DAT Scan and normal/Fluorodopa PET and normal	100	16.4

Red Flags	Sensitivity	Specificity

Rapid progression of Gait impairment requiring wheelchair within 5 years of disease onset	91.2 (85-96)	24.7 (7 – 53)
PD and complete lack of progression within 5 years, unless related to treatment	99.1	ND
Early Bulbar dysfunction, sever dysphonia or dysarthria (speech unintelligible most of the time or severe dysphagia (requiring soft food, NG or PEG within first 5y)	97.4	34.7 (23 – 46)
Inspiratory stridor	99.1	13.9 (0 – 31)
Severe autonomic failure within first 5 years; Orthostatic hypotension (see definition) or	92.6 (90 – 100)	38.3 (0 – 68)
Severe urinary retention /incontinence (associated with erectile dysfunction in men)	100	46.7 (14 – 86)
PD and recurrent falls (>1 per year; how many?) within first 3 years	96.1 (92 – 100)	29.3 (12 – 38)
PD and dystonic anterocollis within first 10 years or contractures	ND	20.1 (9 – 32)-
Absences of: after 5 years of disease onset: sleep disorder: insomnia, RBD, EDS, autonomic dysfunction, hyposmia, psychiatric symptoms: depression, anxiety, hallucinations)	ND	ND
PD and Pyramidal tract signs	92.7 (92 – 93)	32.8 (11 – 52)
Bilateral symmetric Parkinsonism	81.8 (72 – 98)	56.9 (24 – 81)

Data are expressed as Mean % (range) Sensitivity and Specificity.
Specificity is calculated using accumulated total of all atypical parkinsonian syndromes (DLB, MSA, PSPS, CBS) for each criteria
ND – No data

Preferred Reporting Items for Systematic reviews and Meta-Analyses extension for Scoping Reviews (PRISMA-ScR) Checklist

SECTION	ITEM	PRISMA-ScR CHECKLIST ITEM	REPORTED ON PAGE #
**TITLE**			
Title	1	Identify the report as a scoping review.	Not included
**ABSTRACT**			
Structured summary	2	Provide a structured summary that includes (as applicable): background, objectives, eligibility criteria, sources of evidence, charting methods, results, and conclusions that relate to the review questions and objectives.	3
**INTRODUCTION**			
Rationale	3	Describe the rationale for the review in the context of what is already known. Explain why the review questions/objectives lend themselves to a scoping review approach.	4
Objectives	4	Provide an explicit statement of the questions and objectives being addressed with reference to their key elements (e.g., population or participants, concepts, and context) or other relevant key elements used to conceptualize the review questions and/or objectives.	6
**METHODS**			
Protocol and registration	5	Indicate whether a review protocol exists; state if and where it can be accessed (e.g., a Web address); and if available, provide registration information, including the registration number.	21
Eligibility criteria	6	Specify characteristics of the sources of evidence used as eligibility criteria (e.g., years considered, language, and publication status), and provide a rationale.	21
Information sources*	7	Describe all information sources in the search (e.g., databases with dates of coverage and contact with authors to identify additional sources), as well as the date the most recent search was executed.	20
Search	8	Present the full electronic search strategy for at least 1 database, including any limits used, such that it could be repeated.	20
Selection of sources of evidence†	9	State the process for selecting sources of evidence (i.e., screening and eligibility) included in the scoping review.	20

Data charting process‡	10	Describe the methods of charting data from the included sources of evidence (e.g., calibrated forms or forms that have been tested by the team before their use, and whether data charting was done independently or in duplicate) and any processes for obtaining and confirming data from investigators.	21
Data items	11	List and define all variables for which data were sought and any assumptions and simplifications made.	20
Critical appraisal of individual sources of evidence§	12	If done, provide a rationale for conducting a critical appraisal of included sources of evidence; describe the methods used and how this information was used in any data synthesis (if appropriate).	21
Synthesis of results	13	Describe the methods of handling and summarizing the data that were charted.	21

SECTION	ITEM	PRISMA-ScR CHECKLIST ITEM	REPORTED ON PAGE #
**RESULTS**			
Selection of sources of evidence	14	Give numbers of sources of evidence screened, assessed for eligibility, and included in the review, with reasons for exclusions at each stage, ideally using a flow diagram.	21
Characteristics of sources of evidence	15	For each source of evidence, present characteristics for which data were charted and provide the citations.	21
Critical appraisal within sources of evidence	16	If done, present data on critical appraisal of included sources of evidence (see item 12).	21
Results of individual sources of evidence	17	For each included source of evidence, present the relevant data that were charted that relate to the review questions and objectives.	6
Synthesis of results	18	Summarize and/or present the charting results as they relate to the review questions and objectives.	6
**DISCUSSION**			
Summary of evidence	19	Summarize the main results (including an overview of concepts, themes, and types of evidence available), link to the review questions and objectives, and consider the relevance to key groups.	10
Limitations	20	Discuss the limitations of the scoping review process.	16
Conclusions	21	Provide a general interpretation of the results with respect to the review questions and objectives, as well as potential implications and/or next steps.	19
**FUNDING**			
Funding	22	Describe sources of funding for the included sources of evidence, as well as sources of funding for the scoping review. Describe the role of the funders of the scoping review.	2

From: Tricco AC, Lillie E, Zarin W, O'Brien KK, Colquhoun H, Levac D, et al. PRISMA Extension for Scoping Reviews (PRISMAScR): Checklist and Explanation. Ann Intern Med. 2018;169:467–473. doi: 10.7326/M18-0850.
